# Exploration of combined factors related to quality of life after knee replacement surgery

**DOI:** 10.1371/journal.pone.0323007

**Published:** 2025-05-07

**Authors:** Kohki Santoh, Hayato Shigetoh, Hiroaki Yamano, Kohtaroh Torizawa, Hiroshi Takasaki, Daisuke Uritani

**Affiliations:** 1 Department of Musculoskeletal Rehabilitation, Graduate School of Health Sciences, Kio University, Nara, Japan; 2 Department of Rehabilitation, Ichinomiya Nishi Hospital, Aichi, Japan; 3 Department of Physical Therapy, Faculty of Health Sciences, Kyoto Tachibana University, Kyoto, Japan; 4 Department of Physical Therapy, Faculty of Health Sciences, Osaka Yukioka College of Health Science, Osaka, Japan; 5 Department of Rehabilitation, Yamauchi Hospital, Gifu, Japan; 6 Department of Physical Therapy, Saitama Prefectural University, Saitama, Japan; Sheikh Hasina National Institute of Burn & Plastic Surgery, BANGLADESH

## Abstract

In this study, we aimed to identify combined factors associated with lower postoperative quality of life (QOL) in knee replacement (KR) patients, utilising data from the Osteoarthritis Initiative (OAI) database. The data of 44 individuals from the OAI who underwent KR surgery was included in this study. Preoperative baseline data, including demographic information, comorbidities, depressive symptoms, knee-joint symptoms, and health-related QOL, were analysed using association rule analysis to identify single and combined factors linked to low postoperative QOL that were assessed with Short Form-12. Preoperative factors such as comorbidities, high Western Ontario and McMaster Universities Osteoarthritis Index (WOMAC)-pain scores, poor physical function, and older age were strongly associated with lower postoperative physical component scores (PCS) at 2 years. When combined, these factors showed even stronger associations with lower PCS. No significant associations were found with PCS and mental component scores (MCS) at 1 and 2 years postoperatively. Our findings emphasize the importance of evaluating combined preoperative factors, including comorbidities, pain levels, physical function, and age, as they may be associated with lower postoperative QOL in patients who underwent KR. Considering combined factors, rather than assessing single factors in isolation, may provide a more appropriate understanding of postoperative outcomes.

## Introduction

The incidence of knee osteoarthritis (KOA), a degenerative disease characterised by knee stiffness, instability, and pain [1], increases with age. Over 80% of individuals aged ≥60 years exhibit some form of imaging evidence of osteoarthritis, and approximately 40% and 10% of those belonging to this age group complain of symptoms and experience difficulty in daily living, respectively [2].

In patients with KOA, pain and functional disability are strongly associated with lower quality of life (QOL) [3]. While knee replacement (KR) can effectively improve QOL, knee pain, and physical function [4–6], approximately 30% of the patients have reported no improvement in QOL at 1 year postoperatively [7]. Therefore, it is important to consider postoperative QOL from preoperative rehabilitation to postoperative intervention.

Recent reports have identified sex, body mass index (BMI), educational background, knee pain, number of comorbidities, and psychosocial issues as preoperative factors related to postoperative QOL in KR patients [8–10]. Additionally, a systematic review showed that female sex, having fewer comorbidities, and higher BMI were factors contributing to lower QOL after KR surgery; however, the impact on QOL was weak [11].

While recent studies on patients who have undergone KR have largely focused on individual risk factors, a combination of these factors may have a more pronounced effect on postoperative outcomes. Evidence from other clinical studies suggests that even individual factors with weak associations when combined can have significantly stronger associations with adverse outcomes. For instance, a previous study on the development of depression in adolescents revealed that family problems and peer/teacher support were the sole predictors of depression onset and that the combination of these two factors was more strongly associated with depression onset in adolescents [12]. With respect to musculoskeletal disorders, a previous report showed that a lower lumbar muscle activity corresponded to a greater impairment in performance among patients with chronic low back pain and that reduced lumbar muscle activity, combined with pain-related factors (self-efficacy, anxiety, and depression), was more strongly associated with worsening disability than reduced muscle activity alone [13]. Similarly, a single factor weakly associated with postoperative QOL may have a stronger association when combined with other factors.

Nonetheless, the combination of factors strongly associated with postoperative QOL and the difference in the strength of association between single and multiple factors remain unknown. In the present study, we aimed to identify the combined factors associated with lower postoperative QOL in KR patients. We hypothesised that the association with lower postoperative QOL might be even stronger when known QOL-related factors are combined in postoperative KR patients. This study has significance in that, to date, the reported factors associated with worsening postoperative QOL in KR are single factors. Additionally, the results of this study may clarify the composite factors negatively influencing postoperative QOL and provide further insights into the reasons for the decline in QOL.

## Materials and methods

### Participants

The study population comprised 4796 individuals enrolled in the Osteoarthritis Initiative (OAI), which included a database of patients with KOA in the United States. The OAI was a longitudinal study that investigated the onset and progression of KOA and was conducted from February 2004 to October 2015. The OAI was performed in accordance with the principles embodied in the Declaration of Helsinki and was approved by the ethics committees of four institutions (Memorial Hospital of Rhode Island Institutional Review Board, University of Maryland Baltimore—Institutional Review Board, University of Pittsburgh Institutional Review Board, and The Ohio State University’s Institutional Review Board). All participants provided written informed consent before starting the study. A summary of the inclusion and exclusion criteria for study participation in the OAI and the data collected are available at https://data-archive.nimh.nih.gov/oai/. We created our OAI account on 30/01/2018 and obtained the data for research purposes thereafter. The OAI is registered on ClinicalTrials.gov (NCT00080171).

**Fig 1:**
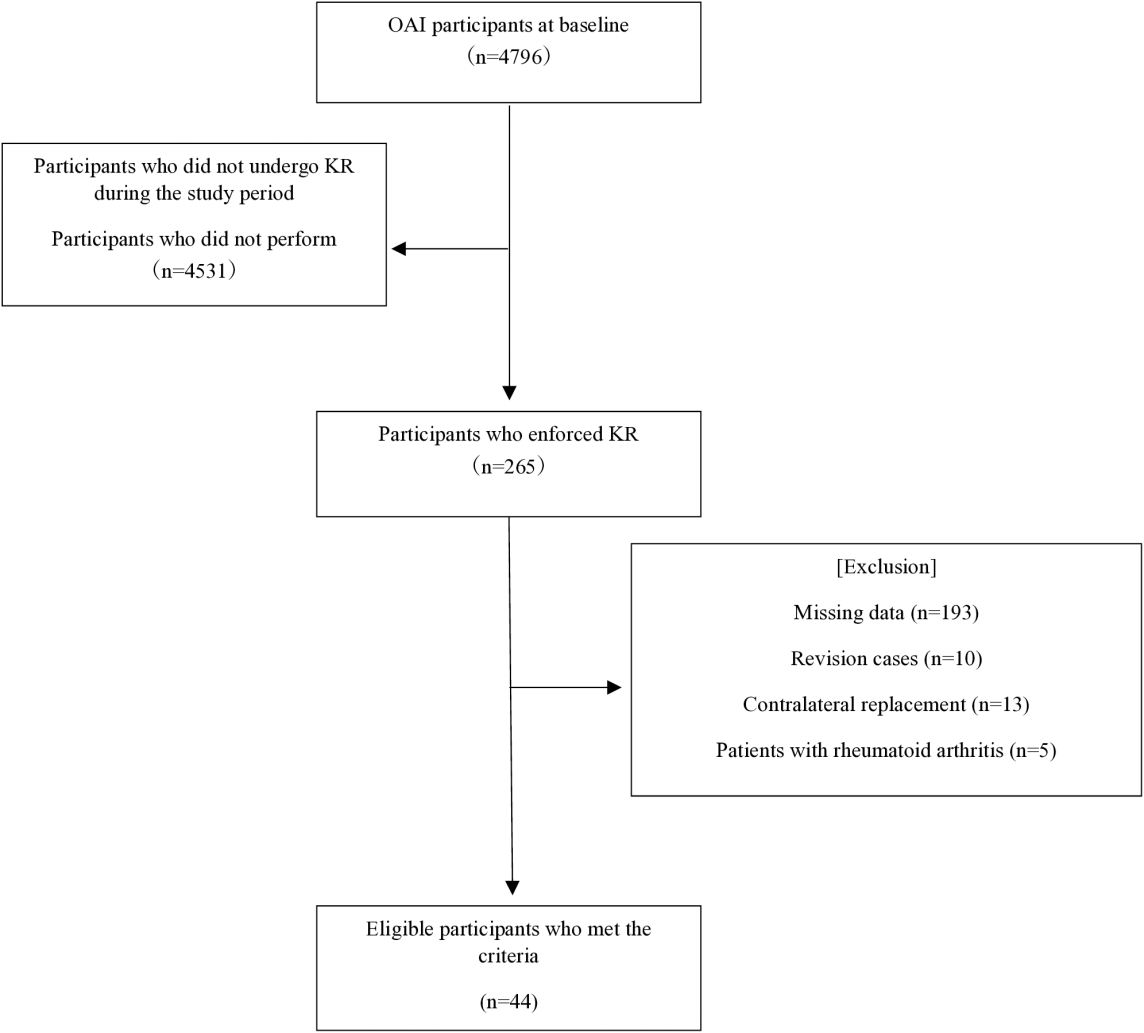
Flowchart of OAI participants showing participant selection. KR, knee replacement; OAI, Osteoarthritis.

The present study included individuals who underwent KR surgery and were traceable from before surgery to 2 years after surgery. The inclusion criteria were as follows: (i) patients who underwent KR on one side and (ii) patients who underwent bilateral simultaneous KR. The exclusion criteria were as follows: (i) revision surgery, (ii) KR performed on the contralateral side during the study period, (iii) rheumatoid arthritis, and (iv) missing data on variables used in the analysis (Fig 1). Preoperative comorbidities have been shown to affect postoperative QOL [11]. This study aimed to explore the comprehensive QOL over time in patients who had undergone KR. Therefore, individuals with comorbidities, such as cancer, stroke, or a history of total hip replacement, were not excluded. Additionally, since dementia was not specifically assessed in the OAI dataset, individuals with dementia were not excluded from this study. The OAI data were stored in a computer protected by security software and passwords and were de-identified when the data were provided by the National Institute of Mental Health so that the individuals could not be identified. This study was approved by the Research Ethics Committee of Kio University (approval no.: R4-13).

### Basic attributes

Preoperative baseline data referred to the data at 1 year before the time when KR was confirmed to have been performed. Sex, age, BMI, sit-to-stand test, comorbidities, Center for Epidemiologic Studies Depression Scale (CES-D) value, and Western Ontario and McMaster Universities Osteoarthritis Index (WOMAC) version LK 3.1 scores were extracted from the baseline data.

### Sit-to-stand test

Impaired performance in standing is linked to factors predictive of KOA progression [14,15]. In this study, physical capacity, including leg strength and knee-joint function, was assessed using the sit-to-stand test. The participants were instructed to rise from a 45 cm chair, cross their arms in front of their chest, and return to the seated position as expeditiously as possible, completing five repetitions. The total time (in seconds) was recorded using a stopwatch, starting with the initial movement of standing on the first repetition and concluding after the return to the seated position on the fifth repetition. The average time of two trials of the sit-to-stand test with five repetitions was used in the current analysis; this test schedule demonstrated high test-retest reliability in individuals with KOA [16,17].

### Depressive symptoms

Depressive symptoms were evaluated using the CES-D [18], a self-administered questionnaire comprising two components (namely, negative and positive) developed to identify depression in the general population. The CES-D exhibits a high level of reliability, with Cronbach’s alpha coefficient of 0.85 in the general population and 0.90 in patients with mental illness. The retest results indicated a moderate correlation, with coefficients of 0.45–0.70. Its high correlation with established tools for assessing depressive symptoms underscores its status as a well-validated questionnaire for evaluating depression [19].

The negative component of the CES-D consists of 16 questions exploring depressive symptoms, physical manifestations, and interpersonal relationships, whereas its positive component comprises 4 questions focusing on positive mood. The severity of depression was examined on a four-point scale: ‘rarely or never’ (less than 1 day), ‘some or a little’ (1–2 days), ‘sometimes or a lot’ (3–4 days), and ‘usually or always’ (5–7 days) (negative: 3 points, positive: 0 point). Weights were assigned to the negative items as follows: 0 for less than 1 day, 1 for 1–2 days, 2 for 3–4 days, and 3 for 5–7 days. Conversely, weights were assigned to the positive items in the reverse order: 3 for less than 1 day, 2 for 1–2 days, 1 for 3–4 days, and 0 for 5–7 days. The scores ranged from 0 to 60 points, with lower CES-D values signifying lower depression levels.

### Comorbidities

Comorbidities were evaluated using the Charlson Comorbidity Index (CCI) [20], an index used to evaluate the presence or absence of 22 diseases, in which a score is assigned to each disease. Myocardial infarction, congestive heart failure, peripheral vascular disease, cerebrovascular disease, dementia, chronic disease, collagen disease, peptic ulcer, and mild liver disease each received 1 point. Hemiplegia (including paraplegia), moderate or severe liver disease, diabetes (with three major comorbidities), solid tumour, leukaemia, and lymphoma each received 2 points. Moderate-to-severe liver dysfunction received 3 points. Metastatic solid tumour and AIDS received 6 points. The higher the total score, the greater the number of comorbidities according to the CCI.

### Knee-joint symptoms

Knee-joint symptoms were assessed using the WOMAC version LK 3.1, and the status of knee symptoms during the past 7 days was evaluated [21]. The WOMAC comprises 24 items grouped into three subscales—namely, knee pain (0–20 points; pain in walking, going up and down stairs, lying in bed, sitting, and standing upright [5 items]); stiffness (0–8 points; stiffness after waking up and late in the day [2 items]); and physical function (0–68 points; going up and down stairs, sitting, standing up, bending, walking, getting in and out of the car, shopping, putting on and taking off socks, getting out of bed, lying in bed, getting in and out of the car/tub, sitting, getting in and out of the bathroom, and performing heavy and light household chores [17 items]), with higher scores indicating more severe symptoms and physical dysfunction [22]. The Cronbach’s alpha coefficients for the reliability of the WOMAC subscales (knee pain, stiffness, and physical function) administered to patients with KOA are 0.91, 0.81, and 0.84, respectively, and retest reliability has confirmed a high internal consistency at 0.86, 0.68, and 0.89, respectively [23]. A previous study confirmed its validity by comparing the WOMAC subscale with the Short Form-36 subscale and reported that knee-joint symptoms tended to worsen in patients with KOA exhibiting lower QOL, suggesting that the WOMAC is a valid questionnaire that captures knee-joint symptoms in patients with KOA [23].

### Health-related QOL

Health-related QOL was assessed using Short Form-12 (SF-12) at baseline and at 1 and 2 years postoperatively [24,25]. The questionnaire consists of multiple questions measuring eight health concepts: (1) physical function, (2) daily role function (physical), (3) bodily pain, (4) overall sense of health, (5) vitality, (6) social life function, (7) daily role function (mental), and (8) mental health. The physical component score (PCS) and mental component score (MCS) are calculated in the range of 0–100, with higher scores indicating a better health status. The Cronbach’s alpha coefficients for the reliability of SF-12 conducted on general healthy participants are 0.87 for the PCS and 0.86 for the MCS, signifying a moderate correlation in the retest results (PCS: 0.79, MCS: 0.59)[26]. The SF-12 has been reported to be a validated questionnaire for QOL assessment, with the PCS and MCS tending to have statistically lower values as the number of comorbidities increased [26].

### Data and statistical analyses

**Fig 2:**
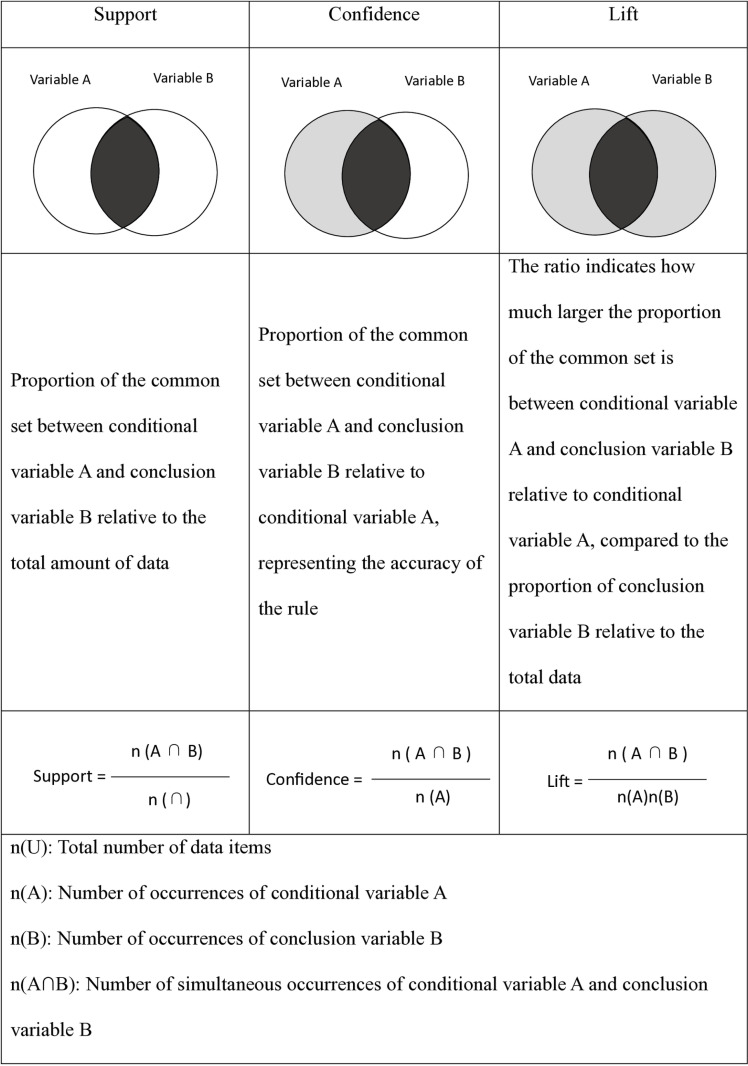
Association rule analysis: support, confidence, and lift.

Association rule analysis extracts correlations between data elements from a large database in the form of rule regularities, ascertains the relationships among the items inherent in the data [27], and evaluates the importance of the extracted rules using support, confidence level, and lift values. Association rule analysis is expressed as A → B, where A is a conditional variable and B is a conclusion variable. The support value indicates the ratio of the amount of common set data for conditional variable A and conclusion variable B to the amount of total data. The confidence level signifies the ratio of the number of common set data items for conditional variable A and conclusion variable B to the number of data items for conditional variable A. The lift value represents the magnitude by which the ratio of the common set for conditional variable A and conclusion variable B to conditional variable A is greater than the ratio of conclusion variable B to the total data. When the lift value is greater than 1, the relationship between A and B is strong, and the occurrence of conditional variable A increases the occurrence of conclusion variable B (Fig 2).

Each variable was classified into two groups based on previous studies or using the current frequency interval method, which is one of the discretisation methods: ‘high-value group’ and ‘low-value group’ (Table 1). For the CES-D, a score of 16 or higher indicated having depressive symptoms [18], age of ≥ 60 years indicated being older [28], BMI of 30 kg/m^2^ [29], or higher indicated obesity, and the presence of one or more comorbidities indicated having comorbidities.

**Table 1:** Data processing methods and cut-off values for each variable.

	Data processing methods	Cut-off value
Age [28]	Cut-off value	60 years
BMI [29]	Cut-off value	30 kg/m^2^
Sit-to-stand test	Equal frequency interval method	–
Comorbidity	Cut-off value	Yes or no
CES-D [18]	Cut-off score	16
WOMAC	Equal frequency interval method	–
PCS and MCS	Standard score	50

BMI, body mass index; CES-D, center for epidemiologic studies depression scale; WOMAC, Western Ontario and McMaster Universities Osteoarthritis Index; PCS, physical component score; MCS, mental component score.

Subsequently, the association rule analysis was conducted to extract the single rule with one conditional variable and the combined rule with two conditional variables, with the PCS and MCS at 1 and 2 postoperative years as the conclusion variables and with the other extracted variables as the conditional variables. The association rule analysis was performed using dummy variables for the conclusion and conditional variables, treating the high-value group as 0 and the low-value group as 1. The minimum thresholds for the extraction of single and combined rules were set at a confidence level of 80% or higher and a lift value of 1.1 or higher [30]. For rules that satisfied the extraction conditions of the association rule analysis, the group that met the rule conditions was classified as the ‘applicable group’, whereas the group that did not meet the rule conditions was classified as the ‘non-applicable group’. Fisher’s exact probability test or χ² test was used to determine whether the ratio of the applicable group to the non-applicable group was statistically significant for the conditional variables [31]. With respect to the combined rule, we examined whether factors meeting the sampling conditions set in the single rule and found to be related in the contingency table test showed higher confidence levels and lift values when combined with other factors, than those in the single rule did. Statistical analysis was performed using R software version 4.2.1 (The R Foundation for Statistical Computing, Vienna, Austria), with the significance level set at 5%.

## Results

### Characteristics of the participants

This study included a total of 44 participants (18 female and 26 male) with a mean age of 64.1 ± 9.8 years. The characteristics of the participants are summarised in Table 2.

**Table 2:** Characteristics of the participants.

Variable	Total data	High group		Low group	
		n	Numerical value	n	Numerical value
Age (years)	64.5 (46.0/80.0)	26	71.5 (62.0/80.0)	18	53.5 (46.0/59.0)
Sex: female/male	18/26	–	–	–	–
Artificial joint type: unilateral/bilateral	41/3	–	–	–	–
Sit-to-stand test	11.4 (6.2/32.7)	22	13.4 (11.5/32.7)	22	9.7 (6.2/11.3)
BMI (kg/m^2^)	29.4 ± 4.3	19	33.4 ± 2.7	25	26.4 ± 2.5
Comorbidity: yes/no	10/34	–	–	–	–
CES-D score	4.5 (0/26.0)	4	22.0 (18.0/26.0)	40	4.0 (0/16.0)
WOMAC: physical function	12.3 (0/41.0)	22	23.4 (14.0/41.0)	22	1.1 (0/10.6)
stiffness	2.0 (0/6.0)	18	4.0 (3.0/6.0)	26	1.0 (0/2.0)
pain	3.0 (0/7.3)	21	8.0 (4.0/15.0)	23	0 (0/3.0)
SF-12 PCS: preoperative	46.2 (15.5/60.5)	17	54.9 (50.2/60.5)	27	39.1 (15.4/50.0)
1 year postoperatively	47.2 (14.0/57.8)	17	52.9 (50.0/57.8)	27	41.5 (14.0/49.7)
2 years postoperatively	43.2 ± 10.4	12	54.9 ± 2.54	32	38.8 ± 8.6
SF-12 MCS: preoperative	55.1 ± 6.6	37	58.1 ± 4.8	7	45.3 ± 3.9
1 year postoperatively	55.3 ± 6.7	31	58.9 ± 3.8	13	46.8 ± 3.3
2 years postoperatively	45.0 (15.3/57.8)	32	59.6 (50.0/66.4)	17	43.8 (33.7/49.9)

Results for each endpoint are expressed as mean ± standard deviation or median (minimum, maximum).

BMI, body mass index; CES-D, center for epidemiologic studies depression scale; WOMAC: Western Ontario and McMaster Universities Osteoarthritis Index; SF-12, short form-12; PCS, physical component score; MCS, mental component score.

### Association rule analysis: single rule

The extracted rules were as follows in a descending order of lift values: comorbidities (one or more), high preoperative WOMAC-physical function score, high preoperative WOMAC-pain score, low preoperative PCS score, poor preoperative standing movement, old age, and high preoperative WOMAC-stiffness score (Table 3). In the contingency table test, the items showing significant differences in proportions between the applicable and non-applicable groups for the conditional variables were comorbidities, preoperative WOMAC-physical function score, high preoperative WOMAC-pain score, low preoperative PCS score, decreased ability to stand, and old age (Table 4). The rule showing a significant difference in the contingency table test results was comorbidities, which achieved the highest confidence level (100%) and lift value (1.38). No single rule for a low MCS at 1 and 2 years postoperatively and no single rule for a low PCS at 1 year postoperatively were extracted.

**Table 3:** Single rule for low physical component score at 2 years postoperatively.

Single rule	Support (%)	Confidence (%)	Lift
Comorbidities	22.73	100	1.38
High preoperative WOMAC-physical function score	45.45	90.91	1.25
High preoperative WOMAC-pain score	43.18	90.48	1.24
Low preoperative PCS	54.55	88.89	1.22
Decreased ability to stand	43.18	86.36	1.19
Old age	50.00	84.62	1.16
High preoperative WOMAC-stiffness score	34.09	83.33	1.15
Low PCS at 1 year postoperatively	50.00	81.48	1.12

WOMAC, Western Ontario and McMaster Universities Osteoarthritis Index; PCS, physical component score.

**Table 4:** Association between the presence of a single rule and low physical component score at 2 years postoperatively.

Variable		PCS at 2 years postoperatively		p-value
		Low group (n)	High group (n)	
Comorbidities	Applicable group	10	0	<0.05
	Non-applicable group	22	12	
High preoperative WOMAC-physical function score	Applicable group	20	2	<0.05
	Non-applicable group	12	10	
High preoperative WOMAC-pain score	Applicable group	19	2	<0.05
	Non-applicable group	13	10	
Low preoperative PCS	Applicable group	24	3	<0.01
	Non-applicable group	8	9	
Decreased ability to stand	Applicable group	19	3	<0.05
	Non-applicable group	13	9	
Old age	Applicable group	22	4	<0.05
	Non-applicable group	10	8	
High preoperative WOMAC-stiffness score	Applicable group	15	3	0.30
	Non-applicable group	17	9	
Low PCS at 1 year postoperatively	Applicable group	22	5	0.16
	Non-applicable group	10	7	

WOMAC, Western Ontario and McMaster Universities Osteoarthritis Index; PCS, physical component score.

### Association rule analysis: combined rule

Table 5 shows the rules that included the rule that had an association with the 2-year postoperative PCS low-value single rule, whereas Table 6 presents the results of the contingency table tests for the association between the presence of combined rule and low PCS at 2 years postoperatively. The low preoperative PCS, high preoperative WOMAC-pain score, high preoperative WOMAC-physical function score, decreased ability to stand, and old age showed higher reliability and lift values when combined with factors other than the single rule. In the contingency table test, the ‘complication + low preoperative MCS’ and ‘complication + decreased ability to stand’ rules were the only rules that did not show significant differences in the ratio of the applicable group to the non-applicable group for the conditional variables. In contrast, other combinations exhibited significant differences in the ratio of the applicable group to the non-applicable group for the conditional variables.

**Table 5:** Combined rule for low physical component score at 2 years postoperatively.

Single factors	Combination factors	Support (%)	Confidence (%)	Lift
Comorbidities	Artificial joint type: unilateral	20.46	100	1.38
	Low preoperative MCS	20.46	100	1.38
	Non-depressive symptoms	20.46	100	1.38
	Decreased ability to stand	18.18	100	1.38
Old age	High preoperative WOMAC-pain score	29.55	100	1.38
	High preoperative WOMAC-stiffness score	22.73	100	1.38
	High preoperative WOMAC-physical function score	34.09	100	1.38
Decreased ability to stand	High preoperative WOMAC-pain score	25.00	100	1.38
	High preoperative WOMAC-stiffness score	18.18	100	1.38
	High preoperative WOMAC-physical function score	29.55	100	1.38
	Old age	34.10	93.80	1.29
	Artificial joint type: unilateral	40.90	90.00	1.24
High preoperative WOMAC-physical function score	Low preoperative PCS	38.64	94.44	1.30
	High MCS at 1 year postoperatively	31.82	93.33	1.28
	High preoperative WOMAC-pain score	38.64	89.47	1.23
High preoperative WOMAC-pain score	High preoperative WOMAC-stiffness score	13.64	100	1.38
	Low preoperative PCS	36.36	94.12	1.29
Low preoperative PCS	Female sex	34.09	100	1.38
	Low PCS at 1 year postoperatively	38.64	89.47	1.38
	Decreased ability to stand	36.36	100	1.23

WOMAC, Western Ontario and McMaster Universities Osteoarthritis Index; PCS, physical component score; MCS, mental component score.

**Table 6:** Association between the presence of combined rule and low physical component score at 2 years postoperatively.

Single factors + combination factors		PCS at 2 years postoperatively		p-value
		Low group (n)	High group (n)	
Comorbidities + artificial joint type: unilateral	Applicable group	9	0	<0.05
	Non-applicable group	23	12	
Comorbidities + low preoperative MCS	Applicable group	1	0	1.00
	Non-applicable group	31	12	
Comorbidities + non-depressive symptoms	Applicable group	9	0	<0.05
	Non-applicable group	23	12	
Comorbidities + decreased ability to stand	Applicable group	8	0	0.08
	Non-applicable group	24	12	
Old age + high preoperative WOMAC-pain score	Applicable group	13	0	<0.01
	Non-applicable group	19	12	
Old age + high preoperative WOMAC-stiffness score	Applicable group	10	0	<0.05
	Non-applicable group	22	12	
Old age + high preoperative WOMAC-physical function score	Applicable group	15	0	<0.01
	Non-applicable group	17	12	
Decreased ability to stand + high preoperative WOMAC-pain score	Applicable group	11	0	<0.05
	Non-applicable group	21	12	
Decreased ability to stand + high preoperative WOMAC-physical function score	Applicable group	13	0	<0.01
	Non-applicable group	19	12	
Decreased ability to stand + old age	Applicable group	15	1	<0.05
	Non-applicable group	17	11	
Decreased ability to stand + artificial joint type: unilateral	Applicable group	18	2	<0.05
	Non-applicable group	14	10	
High preoperative WOMAC-physical function score + low preoperative PCS	Applicable group	17	1	<0.05
	Non-applicable group	15	11	
High preoperative WOMAC-physical function score + high MCS at 1 year postoperatively	Applicable group	14	1	<0.05
	Non-applicable group	16	11	
High preoperative WOMAC-physical function score + low preoperative PCS	Applicable group	17	1	<0.05
	Non-applicable group	15	11	
High preoperative WOMAC-physical function score + high preoperative WOMAC-pain score	Applicable group	17	2	<0.05
	Non-applicable group	15	10	
High preoperative WOMAC-pain score + high preoperative WOMAC-stiffness score	Applicable group	13	2	<0.05
	Non-applicable group	19	10	
High preoperative WOMAC-pain score + low preoperative PCS	Applicable group	16	1	<0.05
	Non-applicable group	16	11	
Low preoperative PCS + female sex	Applicable group	15	0	<0.01
	Non-applicable group	17	12	
Low preoperative PCS + low PCS at 1 year postoperatively	Applicable group	17	2	<0.05
	Non-applicable group	15	10	
Low preoperative PCS + decreased ability to stand	Applicable group	16	0	<0.01
	Non-applicable group	16	12	

WOMAC, Western Ontario and McMaster Universities Osteoarthritis Index; PCS, physical component score; MCS, mental component score.

## Discussion

The present study employed the association rule analysis to explore the combined factors related to poor QOL in 44 postoperative KR patients. No single or combined rule for low MCS at 1 and 2 years postoperatively and no single or combined rule for a low PCS at 1 year postoperatively were extracted. Nevertheless, single rules for a low PCS at 2 years postoperatively was identified; comorbidities (at least one), high preoperative WOMAC-physical function score, high preoperative WOMAC-pain score, low preoperative PCS score, poor ability to get up and move, poor standing movement, and advanced age were extracted as the most common reasons for the low PCS at 2 years postoperatively. The most important factor associated with a low PCS at 2 years postoperatively was comorbidities (at least one). When combined with other factors, low preoperative PCS, high preoperative WOMAC-pain score, high preoperative WOMAC-physical function score, decreased ability to stand, and old age were more strongly associated with a low PCS at 2 years postoperatively than the other factors alone. These combinations had a confidence level of 100% and lift value of 1.38 and were as influential as the single factor showing the strongest association (i.e., comorbidities).

### Comorbidity rule

For comorbidities associated with a low PCS at 2 years postoperatively, the reliability and lift values did not change between the single and combined rules, suggesting the strong influence of a single factor [11]. Nonetheless, the contribution of the number of preoperative comorbidities and other relevant factors to postoperative QOL has not yet been reported. In our study, the combination of comorbidities and unilateral replacements and the combination of comorbidities and non-depressive symptoms were extracted as a combined rule; however, the reliability and lift values did not change with these combinations. Therefore, even in the absence of depressive symptoms or in the case of unilateral replacements, the presence of at least one complication might have a strong influence on postoperative PCS, resulting in a lower PCS.

### Old age rule

Old age, along with each of the WOMAC scores, was strongly associated with a lower PCS at 2 years postoperatively, with confidence levels and lift values increasing from 84.62% to 100% and from 1.16 to 1.38, respectively. Previous studies showed that approximately half or more older patients with KOA had worsening WOMAC-pain, WOMAC-stiffness, and WOMAC-physical function scores [32] and that symptoms of knee OA progressed with age, the onset of rest pain, nocturnal pain, and stiffness lasting longer than 30 min after a period of inactivity [33]. Based on these reports, the symptoms of KOA are closely related to ageing. The results of this study suggest that worsening preoperative knee symptoms (pain, stiffness, and physical function) may lead to worsening postoperative PCS, even in the same age group. Therefore, in addition to age, the clinical symptoms of knee OA in individuals must be considered to improve postoperative PCS in older patients.

### Decreased ability to stand rule

The decreased ability to stand was combined with each of the WOMAC scores, with confidence levels and lift values increasing from 86.36% to 100% and from 1.19 to 1.38, respectively. The stand-up test is considered an indicator of lower-limb muscle strength [15]. Previous reports showed that patients with KOA were more likely to experience quadriceps muscle weakness due to knee pain than the general population [34] and that knee pain status and ease of knee movement were related to quadriceps and hamstring muscle strength, with the group with greater muscle strength exhibiting better daily living and pain scores on the Knee Injury and Osteoarthritis Outcome Score [34]. These reports suggest that lower-limb muscle strength and knee-joint symptoms are related factors. Therefore, patients with KOA may experience a decline in joint function as a result of reduced muscle strength around the knee-joint, leading to a decrease in activities of daily living, thereby negatively affecting QOL. The worsening WOMAC scores and the decline in the preoperative ability to stand among postoperative KR patients may reflect their preoperative condition in postoperative activities, which strongly influenced their postoperative PCS.

### High preoperative WOMAC-pain score rule

The confidence levels and lift values of high preoperative WOMAC-pain score, which was negatively associated with a low PCS at 2 years postoperatively, increased from 90.48% to 100% and from 1.24 to 1.38, respectively, when combined with high WOMAC-stiffness score, which was strongly associated with a low PCS at 2 years postoperatively. As shown by several reports, the greater the knee pain and stiffness, the more they negatively affect preoperative and postoperative QOL [35–37]. A previous study identified knee pain and joint stiffness as the main symptoms of KOA and suggested that they were interrelated in terms of pathophysiology [38]. A qualitative study involving patients with KOA indicated that they perceived exercising with knee pain and moving under painful conditions as both negative, expressing that movements would increase their pain [39]. Under painful conditions, patients with KOA may move their knees less, thereby increasing joint stiffness. Hence, pain and stiffness in patients with KOA may interact with each other and may be important factors with a significant impact on disease progression and patients’ lives.

### Low preoperative PCS rule

A low preoperative PCS was identified as the single factor related to a low PCS at 2 years postoperatively and was more strongly related to a low PCS at 2 years postoperatively when combined with female sex, with the confidence levels and lift values increasing from 88.89% to 100% and from 1.22 to 1.38, respectively. Previous reports showed that female patients with KOA had a lower PCS than their male counterparts and that being female itself was a factor that had a negative impact on postoperative QOL after KR [40,41] because women exhibited a lower pain threshold and tolerance than men [42–44]. Additionally, the tendency to maintain a state of pain has been reported to be involved in worsening PCS [45,46]. Differences in pain sensitivity in female patients with KOA may be related to their sense of physical health. These findings suggest that more attention should be paid to preoperative PCS in women with KOA than in men with KOA.

### Low postoperative MCS rule

No single or combined rule for a low postoperative MCS was extracted in this study. Age, number of comorbidities, and preoperative MCS have been reported as factors predictive of a worse 1-year MCS after KR surgery [47]. Patients with KOA exhibit a declining QOL and an increasing number of comorbidities as OA progresses [48,49]. In this study, only the presence or absence of comorbidities was evaluated, and details regarding the impact of having multiple comorbidities (similar to previous studies) and the impact of the severity of comorbidity are not available [11]. Furthermore, the participants tended to be younger and had higher preoperative MCS than those in previous studies [47]. Additionally, the CCI scores were 0 in 34 patients, 1 in 9 patients, and 2 in 1 patient, indicating that the number of comorbidities in the participants was low. This suggests that few participants were younger and had more comorbidities, which might not have been included as a rule for postoperative MCS. Future studies focusing on different age groups and the degree of comorbidities should be conducted on a broader patient population.

### Study limitations

This study has some limitations. Firstly, studies investigating factors related to postoperative QOL in patients who underwent KR have included at least 200 participants [11]. Thus, considering our small sample size of 44 participants, our results must be interpreted should be interpreted with caution. Association rule analysis is an exploratory method, and it is difficult to determine the appropriate sample size a priori. Consequently, the small sample size in this study increases the likelihood that the discovered rules are due to chance and may limit the reliability and generalizability of the findings. Particularly, the small sample size may have a substantial impact on individual patient results. To examine the generalizability of our findings, we conducted Fisher’s exact test or χ² test. However, we could not completely eliminate the potential impact of the small sample size on our results. Furthermore, the patient population in this study exhibited heterogeneity in terms of background and symptoms. The impact of this heterogeneity on our results warrants further investigation. Future research should aim to include a larger sample size to enhance the reliability and generalizability of the findings. Secondly, a large amount of data was missing during the follow-up period, both preoperatively and at 1 and 2 years postoperatively. Older adults with low health awareness showed a decline in daily living ability and physical function and had a high rate of non-participation in health examinations [50]. It is possible that those who participated in the follow-up study were motivated and that only those with relatively high physical function were included in the study. Thirdly, this was a retrospective database-based study that could not consider psychosocial factors (anxiety and self-efficacy). Given that self-efficacy and anxiety are factors influencing postoperative QOL, a new survey that considers these factors should be conducted in the future [51,52]. Fourthly, it remained unclear whether the type of KR was total or compartment replacement. Because the type of KR also affects the degree of physical function recovery after surgery [53], this may have had an impact on the results of this study. Finally, the timing of both surgery and evaluation differed in each participant because the OAI employed the method of ‘whether KR surgery was performed during the follow-up period from the previous survey’ to determine when KR was performed. Therefore, the length of time between surgery and the first postoperative evaluation may have influenced the results.

## Conclusion

To the best of our knowledge, this is the first study to conduct an association rule analysis to explore the combined factors associated with low postoperative QOL after KR surgery. Low preoperative PCS, high preoperative WOMAC-pain score, high preoperative WOMAC-physical function score, decreased ability to stand, and advanced age were independently associated with a low PCS at 2 years postoperatively and, when combined with other factors, were more strongly associated with a low PCS at 2 years postoperatively than the other factors alone. The results of this study suggest that the postoperative QOL of patients after KR surgery should be evaluated while considering not only the preoperative factors alone but also the participants’ characteristics and physical function from various perspectives to identify factors that worsen postoperative QOL.

## Supporting information

S1 Data Analysis data. (XLSX)
